# Insights into Missense SNPs on Amyloidogenic Proteins

**DOI:** 10.3390/proteomes13040064

**Published:** 2025-12-02

**Authors:** Fotios P. Galanis, Avgi E. Apostolakou, Georgia I. Nasi, Zoi I. Litou, Vassiliki A. Iconomidou

**Affiliations:** Section of Cell Biology and Biophysics, Department of Biology, School of Sciences, National and Kapodistrian University of Athens, Panepistimiopolis, 15701 Athens, Greece; galanfo@gmail.com (F.P.G.); avapo@biol.uoa.gr (A.E.A.); gnasi@biol.uoa.gr (G.I.N.); zlitou@biol.uoa.gr (Z.I.L.)

**Keywords:** single nucleotide polymorphisms (SNPs), amyloids, pathogenic mutations, amyloidoses, amyloid beta (Aβ)

## Abstract

**Background:** Amyloidogenic proteins, a heterogenous group of proteins characterized by their ability to form amyloid fibrils, lead to pathological conditions when they undergo abnormal folding and self-assembly. Missense single-nucleotide polymorphisms (msSNPs) may occur in their sequence, disrupting the normal structure and function of these proteins, pushing them towards amyloidogenesis. **Methods:** A comprehensive dataset of amyloidogenic proteins was created and their msSNPs were collected and mapped on their amino acid sequence. The chi squared test, logistic regression and the bootstrap method were used to ascertain the statistical significance of the results. **Results:** The distribution of pathogenic and benign msSNPs highlighted the predicted amyloidogenic segments as hotspots for pathogenic msSNPs. Analysis of the change in residue properties and pathogenicity status revealed that the substitution of negatively charged residues by any other type of residue tends to be pathogenic. Furthermore, certain substitutions were found to be more likely pathogenic than average. Additionally, a case study of APP, a key protein in Alzheimer’s disease, is used as an example. **Conclusions:** This study will hopefully showcase the importance of amyloidogenic protein msSNPs as well as spark an interest in research of the mechanisms that lead to the formation of amyloid deposits under the scope of pathogenic msSNPs.

## 1. Introduction

Amyloid fibrils are extracellular proteinaceous aggregates of a fibrillar nature with a particular cross-β structure and specific tinctorial properties [1]. Amyloid fibrils are formed through the process of amyloidogenesis when certain soluble proteins and peptides partially or fully unfold, aggregate and self-assemble. These proteins and peptides are referred to as amyloidogenic. Interestingly, several amyloidogenic proteins have been identified as major components of amyloid deposits in a number of human pathological conditions, referred to as amyloidoses. Proteins within this group can vary in their structural characteristics, ranging from well-defined structures to fully or partially intrinsically disordered forms, as well as some with unknown conformation [2]. It is widely accepted that amyloid formation is a general property of proteins and peptides [3] that is modulated by their primary structure [4] and the physicochemical properties of the residues. Amyloidogenesis was shown to be guided by small regions within the proteins [5] known as amyloidogenic determinants or aggregation-prone regions (APRs). These regions remain largely unknown for many of the amyloidogenic proteins, as there is limited experimental data. For this reason, prediction methods are often used to identify APRs. Another key factor in amyloidogenic proteins are the various post-translational modifications (PTMs) that can have a crucial role in protein aggregation [6].

Single-nucleotide polymorphisms (SNPs) are the most common polymorphisms and represent about 90% of known polymorphisms in humans [7]. Each SNP is located at a single site within the genome where there are two or more alleles within a population [8]. SNPs that occur within the reading frame of a protein coding sequence and cause the substitution of the canonical amino acid (aa) are referred to as missense SNPs (msSNPs). Since they are considered more likely to impact the phenotype, they are a point of interest in the research of SNPs and diseases [9].

There are certain types of amyloidoses that are affected by msSNPs located near or within the genes coding for proteins which are deposited in the amyloid fibrils. These polymorphisms may affect amyloidogenesis in a variety of ways, such as modifying the ability of enzymes for post-translational modifications of proteins [10]. The association of polymorphisms with protein aggregation is important evidence that aggregation is a major event in the manifestation of these diseases. Furthermore, the study of these pathogenic polymorphisms can provide critical information on the mechanism and the rate of amyloidogenesis [11,12,13]. Given the high prevalence of amyloid-associated diseases, which affect millions of people each year [14,15] and the crucial role of msSNPs in their onset, this study aims to investigate the residue properties and pathogenicity of msSNPs present in the precursor amyloidogenic protein. For this reason, we examined the correlation between msSNPs, amyloidogenic segments and pathogenesis. Additionally, we explored the significance of changes in the physicochemical properties of aa residues in relation to pathogenesis. Lastly, a case study on msSNPs found in Amyloid-β Precursor Protein (APP) links the distribution and nature of amino acid substitutions to the course of amyloidogenesis.

## 2. Materials and Methods

### 2.1. Overview

**Figure 1:**
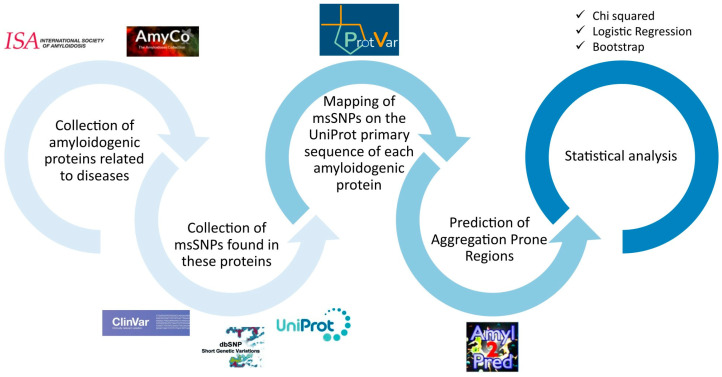
Flowchart of the workflow. First, the amyloidogenic proteins were collected from AmyCo and the list of amyloidogenic proteins by the ISA (2022). The msSNPs found on these proteins were collected from ClinVar, dbSNP and UniProt. The msSNPs were mapped to the primary sequence of each amyloidogenic protein, according to UniProt, thus creating a unified non-redundant dataset. Using AmylPred 2, the APRs were predicted and the msSNPs found within them were collected in the APR-msSNP dataset. Simultaneously, through extensive research of the literature, msSNPs that were related to amyloidoses or diseases related to amyloid depositions were gathered in the disease–msSNP dataset. Statistical analyses were performed; specifically, a chi squared analysis was used to examine the relation between pathogenicity and the location of the substitution in or out of the predicted APRs, logistic regression was performed to examine the relation between pathogenicity and the physiochemical change caused by the substitutions, and bootstrap analysis was used to evaluate the probability that each specific substitution’s association with pathogenicity could arise by chance.

The flowchart of the workflow used is described in Figure 1 and details are provided in the Supplementary File (Methods.pdf).

### 2.2. Amyloidogenic Protein Dataset

The dataset was collected from the International Society of Amyloidosis (ISA) [16] and AmyCo, a collection of amyloidoses and other clinical disorders related to amyloid deposition [17]. The list of human amyloidogenic proteins published by the ISA contains proteins found strictly in extracellular deposits, stated as a major causative factor of each disease. AmyCo focuses on amyloidoses and diseases related to the amyloid deposition. It contains proteins that can be found in amyloid deposits and intracellular amyloid-like inclusions as the major component. AmyCo also contains co-deposited proteins which were excluded from the dataset. Therefore, the dataset contains human proteins that have been identified as the major component of amyloid deposits associated with diseases.

### 2.3. msSNP Dataset

Human genetic variation data were collected from three databases: the UniProt database [18] (release 2022_05 of 14 December 2022), the ClinVar database (release date 5 January 2023) [19] and the dbSNP database (build 155, access date 6 January 2023) [20]. All msSNPs were mapped to the isoform that represents the canonical sequence of each amyloidogenic protein in UniProt, so as to avoid incorrect mapping. For this purpose, the mapping tools ProtVar and Ensembl Biomart [21] were utilized for the mapping of the ClinVar and dbSNP variants, respectively. Substitutions that are not permitted by the genetic code, as they involve more than a single SNP, were removed from the dataset. Similarly, msSNPs that could not be mapped onto the canonical sequence of each protein were also excluded from further analysis. The full list of inclusion and exclusion criteria can be found in the Supplementary File (Methods.pdf).

The final msSNP dataset was created by merging the data collected from all three databases (Supplementary File Data.xlsx Table S1). A common terminology was used for clinical characterization. Three categories were used: pathogenic, benign and unclassified. Thus, msSNPs were labelled as pathogenic if they were characterized as pathogenic or likely pathogenic by UniProt, ClinVar and dbSNP; otherwise, they were labelled as benign if they were characterized as benign, likely benign or protective by UniProt, ClinVar and dbSNP. All other msSNPs were labelled as unclassified. In cases of conflicting clinical significance for the same entry in the databases, the clinical significance from ClinVar was adopted if available; otherwise, the clinical significance of UniProt was used.

The phenotype related to each pathogenic msSNP was then examined as to its relation to amyloid deposition. msSNPs whose related phenotype was included in AmyCo, was an amyloidosis, or was found through a review of the literature to be related to amyloid deposition, were included in a subset of the original dataset, the disease–msSNP dataset (Supplementary File Data.xlsx Table S2).

### 2.4. Prediction of APRs

The effects of msSNPs found within APRs were also studied. To ensure the information processing on amyloidogenic regions was uniform, the consensus algorithm AmylPred 2 [22] was used. AmylPred 2 utilizes 11 methods to predict APRs and has been used effectively by several previous studies [23,24]. For consistent data processing of each amyloidogenic protein, its entire sequence was submitted in AmylPred 2, excluding the signal peptide according to UniProt, when present. The recommended cut-off point of rounding down n/2 methods (5 methods) was used to determine the positive hits. Thus, msSNPs within the predicted APRs were collected in the APRs-msSNP dataset (Supplementary File Data.xlsx Table S3).

Therefore, 2 subsets of the complete dataset were created, the APRs-msSNPs dataset and the disease-msSNPs dataset, containing msSNPs associated with phenotypes correlating with the deposition of amyloid fibrils.

### 2.5. Statistical Analysis

Three statistical analysis methods were applied to confirm the statistical significance of the results. Both the individual aa substitutions and the substitutions grouped in terms of biophysical properties were used for the conduction of additional statistical analyses. Aa residues were categorized as polar, non-polar, positively charged and negatively charged [25].

#### 2.5.1. Chi Squared Goodness-of-Fit Test

To estimate the variance of pathogenic and benign msSNPs of the APRs-msSNPs dataset versus the msSNPs found outside of the APRs, the chi squared test was utilized. This is a statistical test used to examine the differences between categorical variables from a random sample in order to judge goodness-of-fit between observed and expected results [26].

#### 2.5.2. Logistic Regression Analysis

In order to test the association between the biophysical properties of the aa changes (e.g., polar to non-polar) and the pathogenicity status (benign or pathogenic) of msSNPs, logistic regression analysis was performed. Regression analysis can aid the understanding of how the typical value of a dependent variable changes when one of the independent variables is adjusted and the others are held fixed [27].

#### 2.5.3. Resampling with Replacement (Bootstrap)

The bootstrap method was utilized to estimate the statistical significance of each individual aa substitution for benign and pathogenic msSNPs in each subset. Bootstrap is a statistical method that allows us to obtain a theoretical estimate regarding the distribution and the standard deviation of data for a given experimentally obtained dataset (in this case, aa changes) [28]. This method allows a theoretical increase in the available data in a random and unbiased manner [29]. Thus, several “populations” were created, including a pathogenic “population” comprising every pathogenic msSNP found in the complete dataset. Similarly, one pathogenic “population” was created for the disease–msSNPs dataset and one for the APRs-msSNPs dataset. Furthermore, benign “populations” were created for the complete and the APRs-msSNPs datasets. The method was applied 1000 times for each sample, and the average and standard deviation values were determined for all substitutions. The significance of these results was assessed with the calculation of confidence intervals (CIs) for each substitution in all samples. To further determine the significance of each substitution in the pathogenicity status, the odds ratio was utilized [30] in the cases of statistically significant substitutions as derived from bootstrap analysis.

## 3. Results

### 3.1. Amyloidogenic Proteins Dataset

In order to collect the human amyloidogenic proteins, we used the AmyCo and ISA datasets. AmyCo contains well-annotated data for 75 diseases related to the deposition of both intracellular and extracellular amyloid fibrils. In total, it contains 45 proteins that are considered as the major component of the respective amyloid deposits. The list of amyloidogenic proteins from the ISA (2022) contains 42 curated proteins found in extracellular deposits. There is an overlap between both sets and a total of 48 precursor proteins (Table 1) were selected for further analysis, while two proteins and two pharmaceuticals of a proteinaceous nature were excluded. Immunoglobulin light and heavy chains were excluded due to their complex maturation process, resulting in immunoglobulins that differ between patients [31,32]. Enfurvirtide and Glucagon-like peptide 1 analogue were not included in the analysis as there are no corresponding genes in humans.

### 3.2. msSNP Dataset

A total of 15,321 unique msSNPs were collected from dbSNP, ClinVar and UniProt, with 1003 being pathogenic, 495 benign and the remaining 13,823 being unclassified. Pathogenic msSNPs were found in 32 of the proteins and benign in 41; only 2 proteins had exclusively unclassified msSNPs. In the disease–msSNP dataset, 442 pathogenic msSNPs were found in 14 of the proteins. The APRs-msSNP dataset included 230 pathogenic msSNPs found in 23 proteins, 96 benign found in 21 proteins and 2588 unclassified found in 46 proteins. Overall, 120 out of the 230 pathogenic msSNPs were overlapping with the disease–msSNP dataset and were found in 10 proteins (Table 2).

### 3.3. Analyses of msSNP Properties

A number of statistical analysis methods were performed, including the chi squared test, logistic regression and the bootstrap method. These analyses were performed on the full-length precursor (canonical) sequences and did not account for the existence of different proteoforms (e.g., isoforms). Since the total length of the precursor proteins is incomparable to the total length of the amyloidogenic segments, all data had to be normalized based on the length of each segment. It should be noted that unclassified msSNPs are excluded from further analysis as their effects are unknown.

#### 3.3.1. Examining the Distribution of Pathogenic msSNPs Within and Outside of the Amyloidogenic Segments

On average, 7.22 msSNPs per 100 residues were found throughout the length of all proteins and 7.33 msSNPs per 100 residues within the amyloidogenic segments. Pathogenic msSNPs occurred at a rate of 4.84 msSNPs per 100 residues totally and at a rate of 5.17 msSNPs per 100 residues within the amyloidogenic segments. When examining the disease dataset, the rates are 2.13 and 2.70 per 100 residues. Benign msSNPs occurred at rates of 2.39 in total and 2.16 per 100 residues within the amyloidogenic segments. A higher concentration of pathogenic msSNPs and a lower concentration of benign msSNPs was observed in the amyloidogenic segments compared to outside of them. Further statistical analysis was conducted on these results.

First, the chi squared test was performed to examine a possible connection between the position of an msSNP in regard to APRs and its pathogenicity status, with the null hypothesis being that there is no relation between these states. The results of this analysis failed to reject the null hypothesis when examining all msSNPs. However, the analysis of the disease dataset showed a statistically significant (*p* = 0.005) difference in the distribution of the pathogenic and benign msSNPs and their position in relation to APRs.

#### 3.3.2. Examining the Relationship Between the Pathogenicity Status of msSNPs and the Change in Biophysical Properties Caused by msSNPs

**Figure 2:**
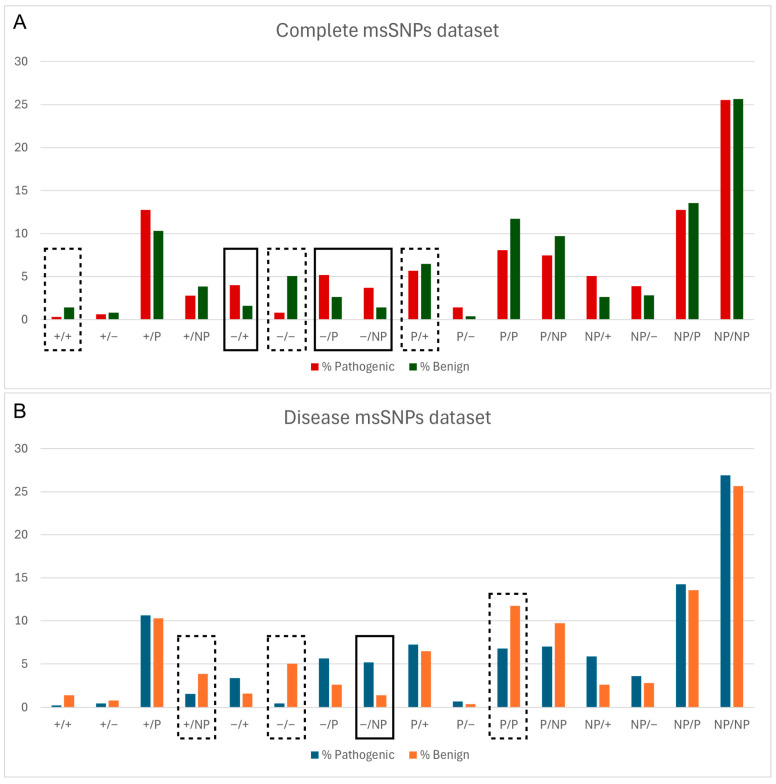
Relative frequency of pathogenic and benign msSNPs, grouped based on the change in physicochemical properties of the aa residues (P—polar, NP—non-polar, ‘+’—positive, ‘−’—negative), both in the complete dataset (**A**) and in the disease dataset (**B**). Statistically significant differences are denoted with a solid line box if the change is more likely Pathogenic, e.g., Negative to Positive (−/+) in (**A**), and a dashed line box if it is more likely Benign, e.g., Negative to Negative (+/+) in (**B**).

Next, the relationship between the pathogenicity status of msSNPs and the change in biophysical properties caused by them was assessed. Logistic regression analysis revealed a connection between the pathogenicity status of msSNPs and the alteration in biophysical properties caused by the residue substitutions. Specifically, msSNPs that caused a shift from a negatively charged residue to any other type of residue were more likely to be pathogenic, while msSNPs that retained the properties of negatively charged residues were shown more likely to be benign (Figure 2).

#### 3.3.3. Examining the Distribution of Residue Substitutions in Relation to the Pathogenicity Status of msSNPs

A more detailed analysis was conducted to examine the relationship of the pathogenicity status and specific residue substitutions. Random sampling with replacement (bootstrap) analysis was employed. This allowed the assessment of the statistical significance of each possible substitution both in the pathogenic msSNPs of each dataset as well as in the benign msSNPs of each dataset. The odds ratio of the pathogenic/benign frequencies of each substitution was used for the statistically significant substitutions.

**Figure 3:**
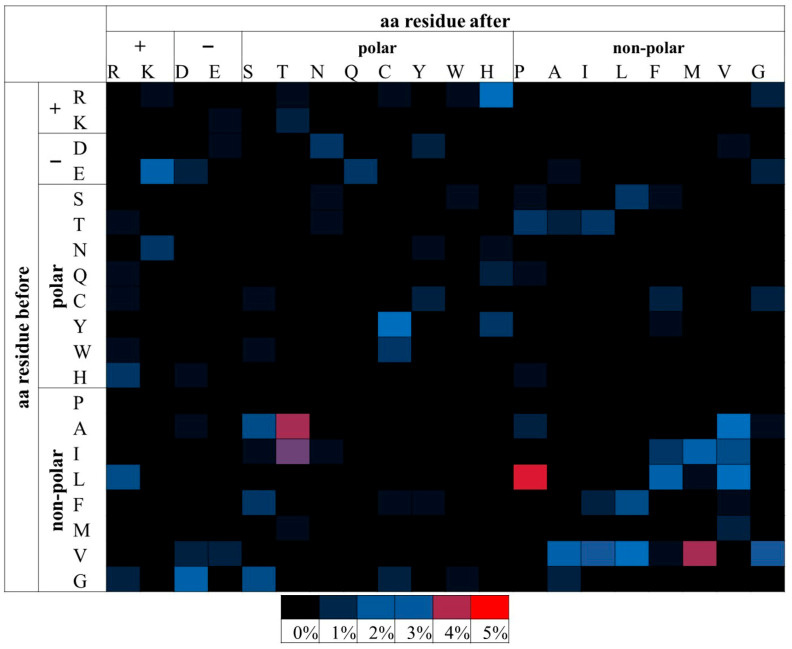
Relative percentage frequencies of pathogenic msSNPs found within the APRs as predicted by AmylPred 2. Aa residues are grouped based on their biophysical properties. msSNPs with higher frequencies tend towards red, mid frequencies towards blue and low frequencies towards black. Only the L→P substitution was found to be statistically significant. It had the highest relative frequency and is marked by red. Other substitutions (such as A→T, V →M and I→T), despite having notably high frequency, were not statistically significant.

From the above analysis, the substitutions of E→K, R→H and L→P were found to be more likely pathogenic when examining all the msSNPs. The substitutions of H→R, R→H and L→P were more likely to be pathogenic, when examining the disease dataset. In the APRs-msSNP dataset (Table 2), only one substitution, L→P (Figure 3), was found to be statistically significant, thus making the L→P substitution the only consistently significant result across the datasets.

### 3.4. Case Study of APP

In order to showcase the impact msSNPs can have on the process of amyloidogenesis, all msSNPs with known clinical significance that are found on Amyloid-β Precursor Protein (APP) were gathered and their distribution and effects were studied. APP is a transmembrane protein implicated in Alzheimer’s disease (AD) and cerebral amyloid angiopathy. APP can be cleaved by β- and γ- secretases, releasing the amyloidogenic peptide Aβ [33]. Several proteoforms of Aβ with varying lengths exist [34] as γ-secretase has multiple cleavage sites, with Aβ_40_ and Aβ_42_ being the most prevalent. While Aβ_40_ is more abundant, Aβ_42_ has been found to form amyloids at a faster rate [35].

**Figure 4:**
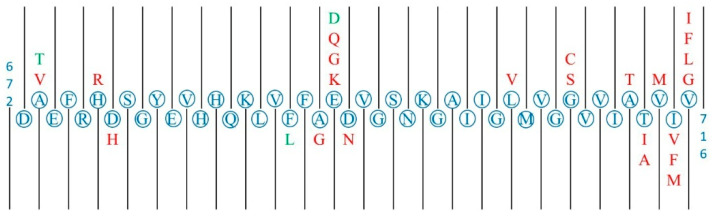
Graphic representation of the section 672–716 of APP which contains the section of the Aβ42 peptide (672–712). The canonical sequence is represented in blue, the pathogenic substitutions are represented in red (i.e., A673V is a pathological substitution) and the benign substitutions are represented in green. The pathogenic substitutions are mainly gathered around three clusters: one near the start of the section and the cleavage site of β-secretase, one near the negatively charged aa residues at the centre of the section and one at the end of the section near several cleavage sites of γ-secretase.

In total, 563 msSNPs were found on the canonical APP sequence (770 aa), 29 of which were pathogenic, 16 benign and 518 unclassified. While the 16 benign msSNPs are dispersed throughout the length of the protein, the pathogenic msSNPs are mostly concentrated in or near the segment of Aβ (Figure 4). Specifically, 12 of the pathogenic msSNPs are located in the segment of Aβ_42_, 10 of the pathogenic msSNPs are located in the four residues following the digestion site of Aβ_42_ by γ-secretase, and the remaining 7 are scattered throughout the length of APP. A protective msSNP is located in the segment of Aβ_42_, specifically the msSNP that causes the substitution of Alanine (A) by Threonine (T) in the position 673 of APP, which is two residues downstream from the digestion site of β-secretase and lessens the affinity of APP to β-secretase, thus causing reduced production of the peptide Aβ [36].

Through extensive literature research, three major ways in which residue substitutions affect the process of amyloidogenesis of the Aβ peptide were found:

- By increasing the affinity of APP and β-secretase, increasing the production of Aβ_40_ and Aβ_42_. The substitutions A2V and A21G follow this mechanism [37,38].
- Increasing the endogenous tendency of Aβ to aggregate into amyloid fibrils. This is how the substitutions E22Q, E22G, E22K and D23N act [39,40,41,42]. It is noted that these substitutions are replacements of a negatively charged residue by a non-negatively charged residue. In contrast, the substitution E22D that maintains the residue charge is characterized as benign.
- Interfering with γ-secretase function, causing an increase in the ratio of Aβ_42_ to Aβ_40_. This is the case for the substitutions T43A, T43I, V44M, I45V, I45F, V46G, V46L, V46F and V46I [11,43,44,45,46,47,48,49,50].

## 4. Discussion

Amyloidogenic proteins are a highly heterogeneous set of proteins that differ in their sequence, structure, function and where they are expressed, but all share the ability to form amyloid fibrils when they misfold and self-assemble. The formed amyloid fibrils are usually associated with pathogenic conditions called amyloidoses. msSNPs are SNPs that occur within the reading frame of a protein coding gene and cause the substitution of a canonical aa residue by a different one. A plethora of msSNPs have been identified on amyloidogenic proteins, with the majority remaining uncharacterized. However, several msSNPs are considered pathogenic, affecting them by pushing them along the pathway that results in their assembly into amyloid fibrils. The ways in which msSNP-induced aa substitutions act on amyloidogenic proteins can vary. It is possible that msSNPs cause protein destabilization and partial misfolding, leading to amyloidogenesis [36,51]. They may also increase the endogenous propensity of the protein to form amyloid fibrils [41,42], or alter the affinity of proteins for post-translational enzymes or other binding sites and as a result promote amyloidogenesis [10,13,52].

From the analysis of the dataset of msSNPs found on human amyloidogenic proteins, it became apparent that about half of the pathogenic msSNPs collected are associated with amyloidoses. At the same time, it was observed that in the predicted APRs the frequency of pathogenic msSNPs, associated with phenotypes correlating with the deposition of amyloid fibrils, is higher compared to the frequency that they occur outside of them. This highlights the importance of these regions in the course of amyloidogenesis, which is further established by the fact that this relation could not be confirmed when examining the complete dataset of msSNPs but was confirmed when examining the disease–msSNP dataset. It must be noted, however, that this study is limited to precursor sequences and thus does not consider the existence of proteoforms with different lengths/sequences. These could alter the significance of msSNPs, such as when they take place in a region not found in the mature amyloidogenic protein (e.g., APP and Aβ).

Furthermore, the statistical study of substitutions at a physicochemical level suggests that msSNPs that cause substitution of negatively charged residues by other residues tend to be pathogenic. Negatively charged residues are known to act as potent inhibitors of amyloidogenesis [53,54,55,56,57]. Additionally, the charge of residues has an important role in stabilizing the normal protein structure and in the interactions with other molecules [58]. The above is further confirmed by the results of the statistical study showing that substitutions that retain the residue charge tend to be benign.

As shown in Figure 2, “Negative to Negative” (−/−) substitutions tend to be benign and “Negative to Non-polar” substitutions tend to be pathogenic; these results were statistically significant and consistent in both the total and disease datasets. In the latter case, not only is an inhibitor of aggregation removed but it is replaced by a non-polar and therefore more aggregation-prone residue. These findings are demonstrated in the case study of APP, where substitutions of negatively charged residues by other residues were pathogenic, including an instance of a “Negative to Non-polar” substitution. On the contrary, the negative-to-negative substitution found in the Aβ sequence was characterized as benign.

In addition, the bootstrap analysis of specific substitutions being pathogenic or benign revealed that the substitution of Glutamic acid (E) by Lysine (K) is most frequently pathogenic when examining the complete dataset. Also, an example of a pathogenic E→K substitution is found in the case study of APP that was shown to increase aggregation [39]. These findings agree with the above results showing the change in charge from negative to positive to be more frequently deleterious. In particular, E has been proven to be an important inhibitor of amyloidogenesis when inserted into amyloidogenic segments [54]. Furthermore, K is a target for more types of PTMs, as well as a more frequently modified site than E [59]. PTMs can have a critical role in amyloidogenic proteins, affecting both their function and aggregation propensity [6], and thereby are also key to understanding disease mechanisms.

When examining the disease dataset, the substitution of Arginine (R) by Histidine (H) and vice versa are the most common pathogenic substitutions. Protein interaction hotspots are enriched in both these residues, especially R [60], therefore such a substitution could lead in a shift in affinity between the amyloidogenic protein and a post-translational enzyme or other ligand. Furthermore, H is the most active versatile residue when it comes to protein interactions as a result of its unique molecular structure and its two possible protonation forms [61], whereas R is a stabilizing factor in the physiological protein structure [62]. Therefore, these substitutions can have deleterious effects on the stability of the protein or its interactions with other molecules. Interestingly, arginine-to-histidine mutations have been associated with cancer and might provide cancer cells an advantage in cases of increased intracellular pH [63]. The protein environment, including factors such as the pH and the net charge, is a determining factor in aggregation [64,65].

The same analysis was repeated for msSNPs within predicted APRs. It was shown that the substitution of Leucine (L) by Proline (P) is most frequently pathogenic. P is a known beta-strand inhibitor due to its unique stereochemistry [66,67] and the predicted amyloidogenic segments often overlap with beta-strands [68]. This suggests that this substitution could act mainly by destabilizing the protein structure, leading to partial unfolding and allowing other amyloidogenic segments to become accessible.

Finally, a case study of the msSNPs found on APP, an extensively studied protein, was performed. Focus was put on the region that contains the Aβ sequence that is relevant to the various proteoforms responsible for aggregation and amyloid plaque formation [34]. The results showed that msSNPs can play a role in the mechanisms of amyloidogenesis in each protein. In the case of APP, it was shown that pathogenic msSNPs mainly affect the affinity of APP with the β- and γ- secretases that are involved in the amyloidogenic pathway or increase the endogenous tendency of Aβ to form amyloid fibrils.

Despite these findings, our study has some limitations. Most polymorphisms have not been characterized and were therefore excluded from most of the analyses. This created issues since, for example, separating the non-polar group into b-breakers and aromatics would require adding several new groups of potential changes (e.g., from aromatics to polar), that would split the dataset into groups that are too small to be statistically analyzed due to the limited data. Hopefully, the results of this study will help emphasize the need for the characterization of msSNPs and potentially guide this process. Also, the use of predicted aggregation regions (AmylPred2) can be considered as a limit but was necessary for data uniformity. Additionally, some key factors in protein aggregation could not be accounted for, such as the effect of the protein environment (temperature, pH, protein charge, etc.) [69]. Furthermore, the analysis focuses solely on canonical protein sequences and not proteoforms (e.g., isoforms, peptides) even though they are important in amyloidogenesis and diseases [6]. For example, as shown in the case study of APP, it is not the precursor protein but the Aβ peptide that forms aggregates composing the AD-related amyloid plaques. Similarly, the other amyloidogenic protein involved in AD, the Tau protein (MAPT), is also found as various proteoforms both due to alternative splicing [70] and to numerous PTMs, primarily phosphorylation but also acetylation, ubiquitination and more, that regulate function and aggregation [71]. Overall, msSNPs are but one factor introducing diversity between proteoforms that act on function and disease along with other factors, such as alternative splicing, proteolysis and PTMs [72]. While the limited availability of data and the enormous complexity of those factors prohibit their exploration by studies like these, they can and should be considered in follow-up studies.

In conclusion, within the framework of this paper a large amount of data on msSNPs in amyloidogenic proteins was collected and processed. Analysis of this dataset could allow better identification of msSNPs with an increased risk of being pathogenic and help with the characterization of the large amount of unclassified msSNPs that were found in amyloidogenic proteins. Moreover, msSNPs were highlighted as a useful tool for exploring the mechanisms and important steps leading to amyloidogenesis in individual proteins, hoping to trigger the study of more amyloidogenic proteins under the scope of the msSNPs found on them.

## Figures and Tables

**Table 1:** Amyloidogenic protein precursors used in this study relative to their source(s).

Protein Name	Source
Apolipoprotein A-I	ISA & AmyCo
Apolipoprotein A-II	ISA & AmyCo
Apolipoprotein A-IV	ISA & AmyCo
Apolipoprotein C-II	ISA & AmyCo
Apolipoprotein C-III	ISA & AmyCo
Amyloid-beta A4 protein	ISA & AmyCo
Beta-2-microglobulin	ISA & AmyCo
Calcitonin	ISA & AmyCo
Corneodesmosin	ISA & AmyCo
Cystatin-C	ISA & AmyCo
Fibrinogen alpha chain	ISA & AmyCo
Gelsolin	ISA & AmyCo
Islet amyloid polypeptide	ISA & AmyCo
Insulin	ISA & AmyCo
Integral membrane protein 2B	ISA & AmyCo
Leukocyte cell-derived chemotaxin-2	ISA & AmyCo
Lactotransferrin	ISA & AmyCo
Lysozyme C	ISA & AmyCo
Microtubule-associated protein tau	ISA & AmyCo
Lactadherin	ISA & AmyCo
Natriuretic peptides A	ISA & AmyCo
Odontogenic ameloblast-associated protein	ISA & AmyCo
Prolactin	ISA & AmyCo
Major prion protein	ISA & AmyCo
Serum amyloid A-1 protein	ISA & AmyCo
Serum amyloid A-2 protein	ISA & AmyCo
Semenogelin-1	ISA & AmyCo
Alpha-synuclein	ISA & AmyCo
Transforming growth factor-beta-induced protein ig-h3	ISA & AmyCo
Transthyretin	ISA & AmyCo
Cathepsin K	ISA
EGF-containing fibulin-like extracellular matrix protein 1	ISA
Pro-glucagon	ISA
Interleukin-1 receptor antagonist protein	ISA
Parathyroid hormone	ISA
Pulmonary surfactant-associated protein C	ISA
Somatostatin	ISA
Transmembrane protein 106B	ISA
Actin, cytoplasmic 1	AmyCo
Actin, cytoplasmic 2	AmyCo
Dysferlin	AmyCo
Huntingtin	AmyCo
Keratin, type II cytoskeletal 1	AmyCo
Keratin, type I cytoskeletal 14	AmyCo
Keratin, type II cytoskeletal 5	AmyCo
Laminin subunit alpha-1	AmyCo
Galectin-7	AmyCo
Superoxide dismutase [Cu-Zn]	AmyCo

**Table 2:** Concise table of the msSNPs according to their clinical significance across the different datasets.

Dataset\Clinical Significance	Pathogenic	Benign	Unclassified	Total
Complete	1003	495	13,823	15,321
Disease–msSNP	442	-	-	442
APRs-msSNP	230	96	2588	2914
Disease and APRs-msSNP	120	-	-	120

## Data Availability

The original contributions presented in this study are included in the article/Supplementary Materials. Further inquiries can be directed to the corresponding author.

## Supplementary Materials

The following supporting information can be downloaded at: https://www.mdpi.com/article/10.3390/proteomes13040064/s1, PDF document: Methods.pdf, Data.xlsx: (Table S1: Complete msSNPs Dataset; Table S2: Disease-msSNP Dataset; Table S3: APRs-msSNP Dataset).

## Abbreviations

The following abbreviations are used in this manuscript:

aa	Amino Acid
APP	Amyloid Precursor Protein
APRs	Aggregation-Prone Regions
ISA	International Society of Amyloidoses
SNPs	Single-Nucleotide Polymorphisms
msSNPs	Missense Single-Nucleotide Polymorphisms
AD	Alzheimer’s Disease
