# Supplementary material for: Insights into Missense SNPs on Amyloidogenic Proteins
Source: Proteomes. 2025 Dec 2;13(4):64. doi: 10.3390/proteomes13040064 (PMC12736960; doi:10.3390/proteomes13040064)
Supplement: Supplementary file 1 [file proteomes-13-00064-s001.zip › Methods.pdf]

Supplementary material

Insights into Missense SNPs on Amyloidogenic Proteins

Fotios P. Galanis, Avgi E. Apostolakou, Georgia I. Nasi [†], Zoi I. Litou, Vassiliki A. Iconomidou *

Section of Cell Biology and Biophysics, Department of Biology, School of Sciences, National and Kapodistrian University of Athens, 15701, Panepistimiopolis, Athens, Greece; galanfo@gmail.com (F.P.G.); avapo@biol.uoa.gr (A.E.A.); gnasi@biol.uoa.gr (G.I.N.); zlitou@biol.uoa.gr (Z.I.L.)

** Correspondence: veconom@biol.uoa.gr*

† Current address: Department of Biotechnology and Biomedicine, Technical University of Denmark, Søltofts Plads, Building 227, 2800 Kongens Lyngby, Denmark.

Detailed methodology

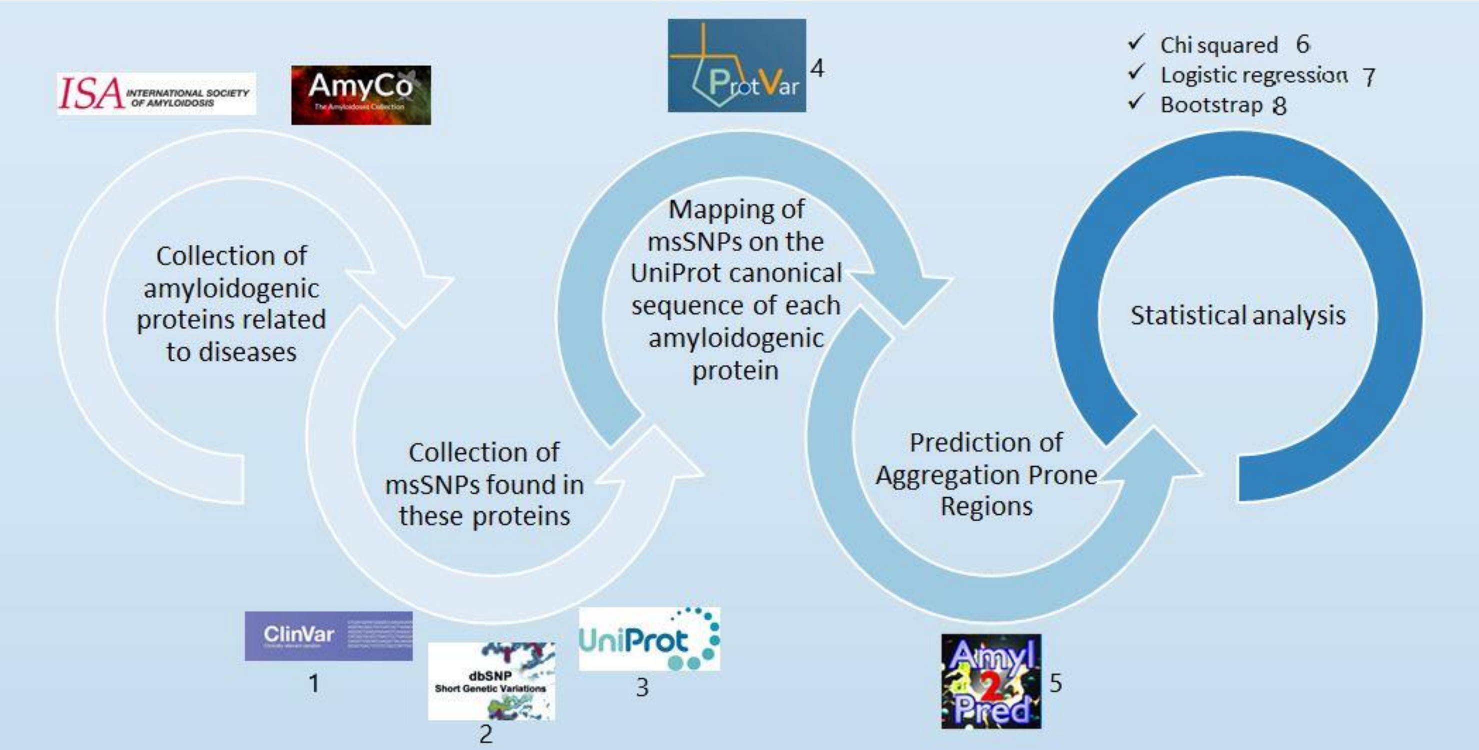

Flowchart depicting the methodology used. Each numbered step is described below.

Description of key steps:

1. Search: ((APP[Gene Name]) OR IAPP[Gene Name]) OR ACTB[Gene Name]) OR ACTG1[Gene Name]) OR APOA1[Gene Name]) OR APOA2[Gene Name]) OR APOA4[Gene Name]) OR APOC2[Gene Name]) OR APOC3[Gene Name]) OR B2M[Gene Name]) OR CALCA[Gene Name]) OR CDSN[Gene Name]) OR CST3[Gene Name]) OR CTSK[Gene Name]) OR DYSF[Gene Name]) OR EFEMP1[Gene Name]) OR FGA[Gene Name]) OR GCG[Gene Name]) OR GSN[Gene Name]) OR HTT[Gene Name]) OR INS[Gene Name]) OR ITM2B[Gene Name]) OR KRT1[Gene Name]) OR KRT14[Gene Name]) OR KRT5[Gene Name]) OR LAMA1[Gene Name]) OR LECT2[Gene Name]) OR LGALS7[Gene Name]) OR LTF[Gene Name]) OR LYZ[Gene Name]) OR MAPT[Gene Name]) OR MFGE8[Gene Name]) OR NPPA[Gene Name]) OR ODAM[Gene Name]) OR PRL[Gene Name]) OR PRNP[Gene Name]) OR PTH[Gene Name]) OR SAA1[Gene Name]) OR SAA2[Gene Name]) OR SEMG1[Gene Name]) OR SFTPC[Gene Name]) OR SNCA[Gene Name]) OR SOD1[Gene Name]) OR SST[Gene Name]) OR TGFBI[Gene Name]) OR TMEM106B[Gene Name]) OR TTR[Gene Name]) OR IL1RN[Gene Name])) AND (("missense variant"[molecular consequence] OR "SO 0001583"[molecular consequence])))) AND "single nucleotide variant"[Type of variation])

2. Queried through BioMart (https://www.ensembl.org/info/data/biomart/index.html)

a. Dataset: Human Short Variants (SNPs and Insertion–deletion mutations (Indels) excluding flagged variants)(GRch38.p13)

b. Filters

- Variant source: dbSNP
- Variant consequences: missense_variant
- Gene stable ID(s): ENSG00000075624,ENSG00000184009,ENSG00000118137,ENSG00000158874,ENSG00000110244, ENSG00000234906,ENSG00000110245,ENSG00000142192,ENSG00000166710,ENSG00000110680, ENSG00000204539,ENSG00000101439,ENSG00000143387,ENSG00000135636,ENSG00000115380, ENSG00000171560,ENSG00000115263,ENSG00000148180,ENSG00000197386,ENSG00000121351, ENSG00000254647,ENSG00000136156,ENSG00000167768,ENSG00000186847,ENSG00000186081, ENSG00000101680,ENSG00000145826,ENSG00000178934,ENSG00000012223,ENSG00000090382, ENSG00000186868,ENSG00000140545,ENSG00000175206,ENSG00000109205,ENSG00000172179, ENSG00000171867,ENSG00000152266,ENSG00000173432,ENSG00000134339,ENSG00000124233, ENSG00000168484,ENSG00000145335,ENSG00000142168,ENSG00000157005,ENSG00000120708, ENSG00000106460,ENSG00000118271,ENSG00000136689

c. Attributes:

- Variant name
- Variant source
- Chromosome/Scaffold name
- Chromosome/scaffold position start(bp)
- Chromosome/scaffold position end(bp)
- Clinical significance
- Phenotype name
- Transcript stable id
- Gene stable id
- Protein Allele
- Variant start in translation (aa)

c.iv. and c.v. was used to exclude any indels (insertion–deletion mutations)

c.viii. Was used to have correct mapping of the substitutions by choosing the transcript that corresponds to the canonical isoform of UniProt

3. The Human Variation Index was used (Release: 2022_05 of 14-Dec-2022) (https://ftp.uniprot.org/pub/databases/uniprot/current_release/knowledgebase/complete/docs/humsa

var.txt)

(At this point 5 Polymorphisms were excluded due to them causing an aa substitution which was not possible to be caused by a single SNP: VAR_016874, rs730881168, VAR_002395, rs886043469, VAR_007597 each of those polymorphisms is the result of 2+ changes in the DNA sequence.)

4. We used ProtVar to correct the mapping for several of the proteins for which the aa substitutions were mapped at a different isoform than the UniProt canonical one. All msSNPs for those proteins (Genes: CDSN, DYSF, GSN, IL1RN, SFTPC, SAA1, FGA, PRNP, MAPT) were put through ProtVar in order to be mapped to the canonical isoform of UniProt. Out of the 1106 msSNPs, only 60 could not be mapped as they were not contained in the transcript for the canonical isoform. At this point a non-redundant dataset was compiled from the results from the three databases.
5. The way that the Clinical Significance was classified can be seen in the following table:

ClinVar	UniProt	dbSNP	Clinical Significance For this study
Pathogenic/Likely Pathogenic	P/LP	Pathogenic/Likely Pathogenic	Pathogenic
Benign/Likely Benign/Protective	B/LB	Benign/Likely Benign/Protective	Benign
Uncertain/Conflicting interpretations	US	Uncertain/Conflicting interpretations	Unclassified

6. From the UniProt data file of each of the amyloidogenic protein we found the Signaling Peptide (if present) and excluded it, then each protein's sequence was examined by AmylPred2 using all 11 methods and the suggested cut-off point of 5 (n/2 rounded down) methods to determine positive hits. Longer proteins had to be "fragmented" into smaller segments (about 200aa each) with overlapping ends as some of the methods are not able to examine long sequences.

7. Chi squared test was conducted to test the hypothesis that the ratio of pathogenic to benign msSNPs within the predicted APRs was higher than outside of them. We used the formula:

$$x^2 = \sum \frac{(Oi - Ei)^2}{Ei}$$

x^2 = chi squared,

Oi = Observed frequency

Ei = Expected frequency

The p value was extracted from a chi square distribution table for 1 degree of freedom

8. Aa residues were grouped into 4 physiochemical categories (Negative, Positive, Polar, Non-Polar)

   SPSS was used to apply the logistic regression analysis. First a table with two columns was created the first column containing the dependent variable (Pathogenicity 0=Benign, 1=Pathogenic) and the second column containing the independent variable (Physiochemical change occurring due to the aa substitution). Binary logistic regression was conducted, the base formula for logistic regression is:

$$z = b_0 + b_1 X_1 + b_2 X_2 + \dots + b_n X_n$$

- z: The measure of the value expressing the total contribution of the participating independent variables (Xi). The value of z is also given by z = log(p/1-p), where p is the probability of the variable being in the state corresponding to 1
- b_0: the slope of the equation equal to the parameter z when all values of the independent variables are equal to zero
- b_i: the regression coefficient or contribution of each independent variable. It indicates how much the dependent variable will change if the independent variable Xi changes by one unit

Since only one categorical independent variable is included in the analysis the polynomial function is of the type $z = b_0 + b_1 X_1$, however, in order to perform the analysis, dummy variables (X1a, X1b etc.) are created as many as the possible states that the variable X1 can take with each dummy variable taking values 0 (if each substitution does not cause the specific change in properties) or 1 (if each substitution causes the specific change in properties).

Of particular interest from the results of the analysis are the significance and the exponential coefficient b (Exp(b)) of each change in the properties of the a.a. Significance is necessary to reject the hypothesis that the differences seen in the pathogenicity-property change relationship are due to a random event, Exp(b) is significant as it reflects the odds ratio of each property change being pathological/neutral, therefore values of Exp(b) greater than 1 indicate a property change that is more often pathological while the opposite is true for property changes with

Exp(b) less than 1.The analysis was conducted with parameters:

```
METHOD=ENTER VAR00002
CONTRAST (VAR00002)=Indicator
ORIGIN
PRINT=CI(95)
CRITERIA=PIN(0.05) POUT(0.10) ITERATE(20) CUT(0.5).
```

8. The resampling with replacement analysis (Bootstrap) was used to identify specific substitutions that were found at a statistically significant frequence, then we used the OR, for substitutions found to have a statistically significant frequence, in order to identify those substitutions that tend to be pathogenic/benign. The process of this analysis was:
   - The creation of each starting "population", meaning that one pathogenic and one benign population was created for the total dataset, likewise we created two starting populations for the APR dataset. For the disease dataset only a pathogenic starting population was created since the benign population is the same for one as the one in the total dataset.
   - Each population was subjected to resampling with replacement through scripts created by us using perl, for the RNG machine we used the Math::Random::MT (https://metacpan.org/pod/Math::Random::MT, seed: 20000) library. 1000 resamples (B) were created for each population.
   - The statistics for each substitution in each population were calculated, thus for each substitution we calculated the mean frequency (μ), the Standard deviation (SD), the Standard error of the mean (SEμ) and the upper and lower Confidence intervals (CI±).
     - $\mu = \sum \quad \chi / B$
     - $SD = \sqrt{\sum \quad (\chi - \mu)2 \,/ B \;\; - 1}$
     - $SE\mu = SD / \sqrt{B}$
     - Confidence Interval 95%, $CI = \mu \pm z_{0,05} \times SE\text{m}$

Where:

- μ is the theoretical average frequency of each amino acid substitution in the resamples
- x is the frequency of the given substitution in each resample
- B is the number of resamples
- SD is the standard deviation
- SEμ is the standard error of mean
- CI is the 95% confidence limit

- z is the critical value for the given confidence level (95%). It is extracted from tables $z_{0.05}$=1.96

- The substitutions for which their actual frequency in the starting dataset was found within their calculated CI± were the ones that were statistically significant.
- For the statistically significant substitutions we calculated their Odds Ratio and the CI± of the OR, substitutions whose OR CIs did not contain 1 were deemed more likely to be pathogenic (if OR>1) or more likely to be benign (if OR<1)
  - $OR = \frac{ad}{bc}$
  - $CI \pm_{OR} = e^{[ln\,(OR) \pm z\sqrt{\left(\frac{1}{a}+\frac{1}{b}+\frac{1}{c}+\frac{1}{d}\right)}]}$

  Where:
  - OR is the Odds Ratio of each substitution
  - a the number of pathogenic msSNPs that cause the specific substitution
  - b the number of benign msSNPs that cause the specific substitution
  - c the number pathogenic msSNPs that cause any other substitution
  - d the number of benign msSNPs that cause any other substitution
  - $CI\pm_{OR}$ The 95% confidence intervals of the OR for the specific substitution
